# Prognosis Prediction in Adult Intensive Care—A Critical Analysis

**DOI:** 10.3390/jcm15208073

**Published:** 2026-10-17

**Authors:** Piotr S. Liberski, Szymon Czajka, Krzysztof Żerdziński, Łukasz J. Krzych

**Affiliations:** Department of Anesthesiology, Intensive Therapy and Perioperative Medicine, Department of Acute Medicine, Faculty of Medical Sciences in Zabrze, Medical University of Silesia in Katowice, 41-800 Zabrze, Poland; pliberski@sum.edu.pl (P.S.L.); szymon.czajka@sum.edu.pl (S.C.); lkrzych@sum.edu.pl (Ł.J.K.)

**Keywords:** prognostication, methods, limitations, critical care, intensive therapy

## Abstract

**Background:** Prognosis prediction in adult intensive care units (ICUs) guides clinical decision-making, realistic goal-setting with families, and resource allocation, but is challenged by the marked heterogeneity of critically ill patients in multi-speciality wards. **Methods:** This narrative review critically analyses commonly used prognostic scoring systems by clinical application timeline. **Results:** Admission prognostic models, including the Acute Physiology and Chronic Health Evaluation (APACHE II/III/IV), Simplified Acute Physiology Score (SAPS II/SAPS 3) and Mortality Probability Model (MPM), estimate in-hospital mortality from acute physiological derangement and comorbidity; the Clinical Frailty Scale (CFS) adds prognostic value. During the ICU stay, organ dysfunction is tracked with the Sequential Organ Failure Assessment (SOFA/SOFA-2), designed for repeated reassessment, and the Multiple Organ Dysfunction Score (MODS), while the Therapeutic Intervention Scoring System (TISS-28) quantifies nursing workload. At discharge, the Barthel Index, Glasgow Outcome Scale (GOS) and Cerebral Performance Category (CPC) measure functional independence and neurological outcome. Admission-based mortality models use an initial assessment window and may require local recalibration, and discrimination alone does not establish clinical utility. **Conclusions:** Future directions include trajectory-based models, phenotyping, biomarkers, time-limited trials and machine learning. Prognostic estimates should inform multidisciplinary evaluation and shared decision-making, rather than independently determine treatment intensity or withdrawal.

## 1. Introduction

Assessing work performance is difficult, as the quantity of work (tasks completed per unit of time) does not always correlate with quality (achieving the intended outcome). This issue is particularly difficult to track and understand within the healthcare system. Health policy has more in common with economics than with the analysis of the safety and effectiveness of medical interventions [1]. Whilst, for example, in the field of cardiovascular diseases, composite endpoints such as major adverse cardiac and cerebrovascular events (MACCE) are used routinely to benchmark the effectiveness of coronary interventions [2], such composite endpoints are in practice defined inconsistently across studies and frequently remain unvalidated [3]—a gap that, if unresolved even in this comparatively homogeneous population, is wider still in intensive care, where the defining problem is instead the marked heterogeneity of critically ill patients treated in multi-speciality (‘medical-surgical’) wards.

Analysing what has happened in the past does not necessarily mean that the same will happen in the future, particularly given medical advances in the fields of diagnosis and treatment. Even the prospective collection of large amounts of information on treatment ultimately provides insight into what has been achieved retrospectively once the database is closed. Consequently, forecasting has become an interesting scientific endeavour. By definition, it is the process of predicting future events on the basis of historical data, relying on current scientific knowledge [4]. The stages of forecasting include: definition (setting the objective and time horizon), analysis (collecting historical data) and modelling (selecting a mathematical method and constructing the model) [5]. The less precisely defined the objective, the longer the time horizon, and the fewer repeatable historical patterns there are, the less certain it is that the system devised will prove successful. Prognosis not only helps clinicians decide on the next course of treatment, but also provides patients and their families with the information needed to set realistic and achievable treatment goals; it is therefore used to determine eligibility for specific healthcare services [6]. This process consists of three elements: assessors use the available tools to estimate the probability of a specific event occurring in a given individual within a specific time frame; the information obtained is communicated to the patient and their family, taking into account the patient’s preferences regarding the scope and manner in which the information is provided; this prognosis is then interpreted by the patient or the person making decisions on their behalf and used in the clinical decision-making process. This approach, utilising a range of diagnostic tools, is successfully applied in oncology and palliative care [7].

There are many mathematical algorithms, mainly scoring systems, which are traditionally used by doctors in intensive care. They can be categorised, depending on the time point of assessment, into those relating to the patient’s condition on admission, during hospitalisation or on discharge (i.e., when are we making the prognosis?). Another classification is based on their purpose: assessing health status, the severity of organ failure, damage to the central nervous system, the degree of disability, workload, or—most commonly—predicting the risk of death (i.e., what are we predicting?). The two classifications do not coincide. Of the instruments discussed, only the admission-based models, namely the Acute Physiology and Chronic Health Evaluation (APACHE), the Simplified Acute Physiology Score (SAPS) and the Mortality Probability Model (MPM), were developed specifically to estimate the probability of death [8,9,10,11]. Organ dysfunction scores, such as the Sequential Organ Failure Assessment (SOFA) and the Multiple Organ Dysfunction Score (MODS), describe the severity and course of organ failure [12,13]; the Therapeutic Intervention Scoring System (TISS-28) measures nursing workload [14]; and the Barthel Index, the Glasgow Outcome Scale (GOS) and the Cerebral Performance Category (CPC) measure functional and neurological outcome [15,16,17]. An association with mortality does not make an instrument a prognostic model in the same sense, and the sections that follow present each instrument in its own role.

The aim of this paper is to present the prognostic scales most used in intensive care and to provide a critical analysis of their potential limitations.

## 2. Materials and Methods

This is a narrative review: no systematic search protocol, database list or search end-date is registered, and no formal risk-of-bias assessment or quantitative synthesis was performed. Sources were identified through database searching and citation tracking from key papers, and the selection of instruments and supporting literature reflects the authors’ clinical and methodological judgement rather than a pre-specified systematic protocol. Priority was given to the original derivation and validation studies of each instrument, to subsequent independent external validations, and to methodological literature on the appraisal of clinical prediction models. Instruments were included if they are in routine adult intensive care unit (ICU) use in Europe and address at least one of the three time points that structure this review (admission, ICU stay, and discharge). Within this frame, the core set comprises the scales recommended for adults in the Polish guidelines on admission to anaesthesiology and intensive care units (APACHE II, SAPS II and SOFA) [18], their successors and principal alternatives (APACHE III and IV, SAPS 3, MPM, SOFA-2 and MODS), the workload index adopted by the public health insurance fund in Poland (TISS-28; Section 4), and established functional and neurological outcome measures (Barthel Index, GOS and CPC). The Clinical Frailty Scale and the Functional Status Score for the ICU were added because they capture pre-admission frailty and in-ICU function, which the core set does not, and the Katz Index as the closest alternative to the Barthel Index; paediatric and disease-specific scores were considered only where they illustrate a general methodological point. This selective, judgement-based approach is a limitation inherent to the narrative review format.

### Use of Generative Artificial Intelligence

During the preparation of this manuscript, generative artificial intelligence was used in a strictly assistive, post hoc verification capacity. After the authors had completed their own drafting and revisions, the manuscript underwent six further rounds of independent critical review by large language models (Claude, Opus 5, Anthropic; ChatGPT, GPT 5.6 Sol, OpenAI), with each round cross-validating the suggestions produced in the previous one across models rather than relying on a single source. In each round, every factual and numerical claim in the text was checked against the full text of the cited primary sources with the explicit aim of catching what the authors themselves might have overlooked: misattributed or substituted references, statements that overstated or understated what a cited study actually reported, and inconsistencies between sections. Every AI-suggested correction was then independently verified against the primary literature and accepted, modified, or rejected by the authors, who take full responsibility for the final content. Generative AI tools were also used for language editing (grammar, spelling, and punctuation) of the English text. The graphical abstract was produced with the assistance of Gemini 3.1 Pro (Google) from an author-prepared brief. No data were fabricated, and no AI output was incorporated without author verification.

## 3. Scales Used When Admitting Patients to the Ward: Mortality Prediction Models and Frailty

The decision to admit a patient for treatment in the anaesthesia and intensive care unit (ICU) is preceded by a near-certain assumption that the implementation of procedures and methods reserved exclusively for that unit will bring a tangible benefit to the patient [18].

When deciding whether to admit a patient, the following factors should be taken into account: the immediacy and severity of the threat to life and health (i.e., organ failure or dysfunction, the severity or stage of the condition, and organ damage); the reversibility of the disease process; the presence of comorbidities; and the results of additional investigations (imaging, laboratory tests, etc.). In some clinical cases, reaching a decision may be difficult. It is not recommended to admit patients to the ICU based solely on numerical data or prognostic scoring systems, which are merely advisory in nature. The recommended scales for adults are: APACHE (Acute Physiology and Chronic Health Evaluation) and SAPS (Simplified Acute Physiology Score). The severity of the patient’s condition on admission to the ward is one of the most important predictors of death.

The original version of the APACHE scale was developed in 1981 by Knaus et al. [19]. It is a mathematical scale that converts scores into probabilities of death, developed based on a historical cohort. It was devised using comprehensive data from just 805 ICU admissions in the United States. However, a modified version, namely the APACHE II scale, has come into use in international practice [8], which includes 12 physiological parameters, age and the presence of selected chronic conditions, including immunocompromise; these chronic health points are weighted by admission type, scoring 5 points for non-operative or emergency postoperative admissions and 2 points for elective postoperative admissions. It takes into account the worst physiological readings over a 24 h period. The complete data used to develop this algorithm were collected from 5815 patients across 13 hospitals. The score correlates non-linearly with the risk of death. For example, a score in the range of 20–24 indicates a 40 per cent risk of death for ‘medical’ patients and a 30 per cent risk for ‘surgical’ patients [20]. Subsequent editions of the scale (III and IV), although much better prepared in methodological and statistical terms, failed to gain popularity, in part because the APACHE III publication withheld its full predictive equation and coefficients as the proprietary intellectual property of APACHE Medical Systems Inc., a decision that provoked editorial controversy and fragmented the field into competing systems [21,22,23].

The original version of the SAPS scale was published by Le Gall et al. in 1984, based on a cohort of 679 patients treated in French ICUs, as the European equivalent of the APACHE scale [24]. It was based on 14 variables describing the worst values measured over a 24 h period. However, in the scientific community, the best-known version is SAPS II, which was developed and validated on an international cohort of over 13,000 critically ill patients. It is based on an assessment of 12 physiological variables, age, type of admission (internal medicine, elective surgery or emergency surgery) and three variables relating to comorbidities (AIDS, haematological malignancies and metastatic cancer) [9]. The total score obtained (the sicker the patient, the higher the score) is converted into a probability of death (in %) using a mathematical formula. In 2005, the SAPS scale was updated to its third version (SAPS 3), which is based on the assessment of 20 variables grouped into three boxes (pre-admission status, circumstances of admission, and acute physiological derangement within the first hour), which resolve into 61 item classes in the final model—pre-admission status alone accounting for half of the model’s explanatory power (50% of Nagelkerke’s R^2^), more than the acute physiology box (27.5%)—and was developed using a cohort of nearly 17,000 patients treated in 2002 [10]. A comparison of the variables entering APACHE II, SAPS II and SAPS 3 is given in Table 1.

The APACHE II, SAPS II and SAPS 3 scoring calculators are available in a publicly accessible version [27].

Over the years, the accuracy of these predictive scales has also been assessed in a population of critically ill patients treated in Polish ICUs [28,29,30] stating that they may be useful, though not perfect, tools for predicting early death, but are of substantially reduced use in assessing the risk of death at a later stage: discrimination for ICU mortality (area under the receiver operating characteristic curve, AUROC, 0.79–0.84 for SAPS II, APACHE II and SOFA) collapsed to 0.66–0.71, still better than chance but of limited clinical value, for mortality after discharge in the same cohort [30].

As regards the possibility of using other scales when admitting patients to the ICU, the CFS (Clinical Frailty Scale) is worth mentioning [31]. This scale provides a clear illustration of the severity of a patient’s functional impairment. Level 1 describes individuals who are fully functional, physically and cognitively active, and energetic; level 7 describes patients who are completely dependent for personal care but not otherwise close to the end of life; levels 8 and 9 (“very severely frail” and “terminally ill”) were added in a later revision of the original 7-level scale, with level 9 specifically denoting a life expectancy of approximately 6 months or less, a category that can apply even to patients who are not otherwise severely frail. Patient classification is carried out holistically as a clinical judgement, informed by overall comorbidity burden and functional trajectory rather than by specific laboratory thresholds. The FSS-ICU (Functional Status Score for the ICU) is also a useful tool for assessing functional status [32]. Frailty, or even a poor quality of life, correlates very strongly with the risk of death [33]. In a cohort of elderly ICU patients, a CFS score of 7 or more was associated with a 2.5-fold higher risk of ICU death (AUROC 0.74).

Another scale is the MPM (Mortality Probability Model), which, like the APACHE and SAPS scales, comprises a set of variables relating to acute conditions and selected chronic diseases. The most widely used version of the MPM was developed in 1993, based on data from a cohort of nearly 20,000 patients from 12 countries [11]. Importantly, burn patients and those admitted for coronary care or after cardiac surgery were excluded from the analysis. Prediction of death on this scale may be based on data recorded on admission or 24 h after admission; 48 and 72 h versions were developed separately by the same investigators using comparable cohorts [34].

## 4. Scales Used During a Patient’s Stay in the Ward: Organ Dysfunction and Workload

During a patient’s stay in the ICU, their current condition is most commonly assessed using a system that analyses the nature and severity of multi-organ failure (MOF). This enables the assessment of trends in changes related to the response to treatment and the risk of an unfavourable outcome [35,36,37]. Organ dysfunction scores may also be calculated on admission to the ICU (usually from the most severe impairment over the first 24 h), and the admission value is associated with the risk of death [37]; they nevertheless remain descriptors of organ dysfunction rather than mortality prediction models [12].

The most commonly used scale is the SOFA (Sequential Organ Failure Assessment) scale [37], which was developed in 1996 as a tool to describe, not predict, organ dysfunction over time; the original consensus paper explicitly cautioned against using it to estimate an individual patient’s risk of death, referring to it instead as the Sepsis-Related Organ Failure score [12]; however, validation studies carried out in 40 ICUs across 16 countries have shown that the scale has good diagnostic accuracy in other groups of critically ill patients [38]. Over the years, numerous publications have confirmed the usefulness of the SOFA scale, which led to the development of a newer version, namely the SOFA-2 scale [39]. The SOFA-2 project covered 1319 ICUs across 9 countries on 5 continents, using data collected between 2014 and 2023. The score was developed and internally validated on four cohorts totalling approximately 2.1 million patients, and externally validated on six further cohorts comprising more than 1.2 million patients. Despite this scale of validation, SOFA-2 improved discrimination for ICU mortality only marginally over the original score (AUROC 0.79, 95% CI 0.76–0.81, versus 0.77, 95% CI 0.74–0.81)—a modest gain consistent with the development protocol, in which content validity took precedence over predictive validity: the score was redesigned to better describe the distribution of organ dysfunction, not to outperform its predecessor as a mortality predictor. Reclassification between the two versions was nonetheless substantial: in the eICU cohort, 49% of patients retained the same total score under SOFA-2, while 40% scored lower and 11% scored higher, with associated ICU mortality of 4.7%, 8.6% and 13.5% respectively. Table 2 compares the organ-system domains and variables used by the two versions.

Alternatively, the MODS (Multiple Organ Dysfunction Score) scale, developed in 1995, can be used [13], which assesses the failure of six vital systems: the respiratory system (PO_2_/FiO_2_ ratio), the circulatory system (heart rate, CVP/MAP ratio), the kidneys (creatinine), the liver (bilirubin), the coagulation system (platelet count) and level of consciousness (GCS score). The failure of each system is scored from 0 to 4. A score of 13–16 predicts that 50 per cent of patients will die in the ICU and approximately 70 per cent in hospital, with a mean ICU stay among survivors of approximately 17 days; a score of more than 20 predicts an ICU mortality rate of 100 per cent. These figures are based on the maximum (worst) score recorded at any point during the ICU stay, derived from a single-centre surgical cohort of 692 patients, and require external validation before being applied to other populations.

When analysing the scales used during a patient’s ICU stay, one cannot fail to mention the Therapeutic Intervention Scoring System (TISS-28), which was designed to assess the workload of ICU staff. Created in 1996, this scale is a shortened version of a scale from the 1970s and contains a list of the 28 most common tasks performed in the ICU, along with assigned scores (ranging from 0 to 78), which are intended to reflect the workload of staff caring for a patient [14]. The scale has been adopted by the public health insurance fund in Poland; however, it does not reflect the realities of modern intensive care.

## 5. Scales Used on Discharge from the Ward: Functional and Neurological Outcome

Predicting patient outcomes upon discharge from the ICU has not become standard practice. However, we often attempt to assess the extent of impairment caused by a critical illness. This correlates directly with the feasibility and appropriateness of implementing rehabilitation, recovery and convalescence programmes targeting motor, cognitive, intellectual, social and occupational functioning.

The most common form of assessment is the scale developed in 1965 by Mahoney and Barthel (BI, Barthel Index) [15]. It is primarily used to assess a patient’s suitability for rehabilitation centres or residential care. The Barthel Index assesses a patient’s functional ability and independence in 10 basic activities of daily living. A patient can score a minimum of 0 and a maximum of 100 points on this scale [40]. Scores of 60 or below indicate severe or total dependency (Table 3). The Katz Index (ADL, Activities of Daily Living) is very similar in structure [41]. It was developed in 1963 and assesses independence in performing the six basic activities of daily living (bathing, dressing, using the toilet, moving about, elimination, and eating). If a patient is independent in all activities, they are assigned category A; if dependent in all, category G. It has been documented that the more impaired a patient is on discharge from the ICU, the poorer their prognosis [42]. The Barthel Index scoring criteria and interpretation bands are summarised in Table 3.

When predicting treatment outcomes in the ICU, it is very common to take into account not only whether a patient has died or survived, but also their neurological function. Originally, such assessments were directed mainly at patients who had suffered a cardiac arrest, but over time they have become more widespread amongst the wider population of critically ill patients. In this context, the literature contains studies utilising the GCS (Glasgow Coma Scale), CPC (Cerebral Performance Category) and GOS (Glasgow Outcome Scale) scales. Of these, the GCS and GOS were originally developed for patients with severe brain injury, while the CPC was developed specifically for patients following cardiac arrest [16,17,43,44]; however, the degree of neurological impairment, regardless of its aetiology, correlates well with a poor prognosis [45]. Table 4 compares the two scales; note that they run in opposite directions—a score of 1 denotes the best outcome on the CPC but death on the GOS—so the two should never be compared or pooled numerically.

## 6. Limitations of Prognostication

Before turning to the specific limitations of individual scales, it is worth distinguishing two independent ways in which a prognostic model can fail. Discrimination, usually reported as the AUROC, describes how well a model separates patients who die from those who survive. Calibration describes whether the predicted probability corresponds to the observed frequency of the event: a score may discriminate acceptably while overestimating absolute risk, and calibration, rather than discrimination alone, is the property that matters when a predicted mortality is discussed with a family. Most published validations of ICU scores report discrimination alone; calibration, when assessed, is typically summarised by the standardised mortality ratio (observed-to-expected deaths), and it may deteriorate when a generic score is applied outside its derivation population. Polish ICU data illustrate both this pattern and the difficulty of interpreting it: a region-wide registry reported observed ICU mortality slightly below that predicted by APACHE II (observed-to-predicted ratio 0.93, predicted mortality 47%) [28], while a separate single-centre Polish cohort reported that APACHE II retained good discrimination for ICU mortality (AUROC 0.836) [30]. Two caveats apply to figures of this kind: a single summary ratio does not describe calibration across the full range of predicted risk, and a ratio that sets observed ICU deaths against a model’s predicted in-hospital mortality compares two different endpoints, and is therefore not by itself evidence of miscalibration for the endpoint the model was built to predict. A further consequence follows: these models were developed and validated primarily to compare cohorts, for benchmarking, case-mix adjustment and research stratification, and are less suited to quantifying risk in an individual patient. Even when a model is well calibrated, a predicted mortality of 90% means that approximately one in ten such patients would be expected to survive, and a score alone cannot identify which one. When a score overestimates risk, quoting its predicted mortality to a family as an individual forecast overstates the threat the patient faces. Prognostic information is better conveyed clearly and without jargon, together with its uncertainty and the factors on which the estimate rests, bearing in mind that families weigh the clinician’s estimate against their own observations and beliefs [6].

A further and less tractable problem affects any prognostic model developed from routinely collected ICU data: predictions of a poor outcome can influence the decisions that generate that outcome. Where life-sustaining therapy is withdrawn because the prognosis is judged hopeless, the resulting death may make the prediction appear confirmed. This circularity is particularly relevant in neuroprognostication after cardiac arrest, where withdrawal of life-sustaining treatment accounts for up to 80% of deaths and where the variables used to predict outcome may also inform the withdrawal decision [46]. Retrospective data alone cannot fully separate this bias from true prognostic accuracy; prospective designs in which prognostic information is blinded to the treating team, or in which withdrawal is deferred for a defined period of full treatment, are better placed to do so [46]. This is also one of the arguments for structuring uncertain cases as an explicit time-limited trial rather than as a single prognostic judgement made at admission [47].

Predicting outcome in the ICU requires knowledge, clinical experience and sound judgement. The systems outlined above are subject to a significant number of limitations [48,49,50]. Their use without critical evaluation—that is, without an understanding of the scale’s underlying principles, its interpretation, or consideration of confounding variables—may lead to estimation errors and limit the conclusions that can be drawn. Models for assessing a patient’s condition severity should be used with an understanding of the strengths and weaknesses of estimating the probability of death in the ICU. It should also be noted that prognosis should not deprive a patient of the opportunity for treatment in the ICU. A scoring system determining the degree of organ failure, comorbidity burden and severity of condition is only one element in assessing a patient’s chances of survival. A high score should prompt earlier and more explicit discussion of goals of care with the patient and family—but it does not, by itself, justify withholding or de-escalating therapy. It may form part of the decision to initiate a time-limited trial (TLT). The analysis of the patient’s clinical situation should be thorough and conducted by a multidisciplinary team. Prognostic scales have been reported to overestimate mortality in a number of validation settings [28,51,52].

The simpler the scale is in its design, the more readily and widely it will be used; however, one must bear in mind that such screening is not very accurate and will usually require a more detailed analysis (as is the case, for example, with laboratory diagnostic tests). In a population of critically ill patients, so many variables influence the prognosis that a single-time-point assessment is unlikely to capture it fully. More important than the condition in which a patient is admitted to the ICU is whether they are making progress because of the treatment administered, and whether complications or adverse medical events have occurred. When a patient is admitted to the ICU, there is often a lack of information regarding their pre-admission health status, and we can never be certain that the variable considered to be the worst is so (sampling frequency may affect which value is recorded as the worst). Some information cannot be reliably assessed, yet its accurate evaluation is crucial for model development [53]. Across 56 published ICU mortality models, the definition of a predictor variable was reported clearly in only 74% of cases and its method of measurement in just 70%; even the ‘worst value’ used by SOFA is defined inconsistently across studies. For example, neurological status may be masked by the effects of sedative drugs; most of these scales do not take into account circulatory support with vasoactive agents other than catecholamines, VIS (vasoactive-inotropic score) values, or coagulation disorders other than those reflected by the platelet count. SOFA-2 is a partial exception: it scores the last Glasgow Coma Scale recorded before sedation, any additional vasopressor or inotrope, mechanical circulatory support and renal replacement therapy [39]. Furthermore, the assessment of a patient’s condition is usually performed at a single point in time; a reliable assessment of prognosis requires insight into the dynamics of changes [54,55]. Furthermore, the scales presented are based on historical data and do not take into account the variation in the characteristics of patients treated in the ICU, which affects their reliability [56,57,58,59]. Finally, the scales in question are not usually validated (calibrated, adjusted) in local conditions, which limits their transferability [60,61].

## 7. Areas for Further Research

It seems that research should soon provide an answer to the question of to what extent it is worthwhile to predict a patient’s outcome using rather archaic tools, and to what extent it is necessary to develop new ones. It would be interesting to determine how the ability to predict death during hospitalisation changes over time. Does the initial assessment correlate with the assessment at discharge? Does the changing patient population over the years alter the rules of prediction? How can prediction be improved by tracking trends in these changes? Finally, we must ask ourselves to what extent such mathematical considerations should influence clinical decisions?

In recent years, much attention has been devoted to patient profiling and phenotyping, based on their individual demographic and clinical characteristics, and possibly genetic predispositions. This is intended to help create specific roadmaps that will enable the precise planning of diagnostic and therapeutic management for a particular case. The aim is to improve not only the accuracy of prognosis, but ultimately outcomes such as length of hospital stay, recovery and quality of life; it should be stressed, however, that better prediction has not yet been shown to translate into better outcomes [62].

An important element of profiling is the identification and monitoring of changes in biomarkers. A biomarker is any objectively measurable biological indicator—most commonly a gene, a substance or a physiological parameter. Today, we know most about the potential for neurological prognosis based on CT (or MRI) scans of the brain, EEGs, pathological neurological symptoms and biomarkers of neuronal damage [46,63,64]. Neurofilament light, for example, predicted poor neurological outcome after out-of-hospital cardiac arrest with 90% sensitivity and 100% specificity at a cut-off of 173.7 pg/mL measured 12 h after return of spontaneous circulation; this figure comes from a pilot cohort of 22 patients, and both the threshold and its timing await confirmation in larger, prospective studies.

Prognostic scales, when combined with other reliable assessment tools, will be used increasingly widely in consultative decision-making regarding the initiation, continuation or discontinuation of treatment when it becomes futile. In intensive care, particularly regarding patients classified as ‘Priority 3’, prognosis may form part of a time-limited trial (TLT). Decisions regarding the limitation or withdrawal of treatment are grounded in medical ethics and address a key issue in intensive care medicine, namely the question of individual benefits to the patient [47]. The benefit to the patient does not always go hand in hand with the benefit to the healthcare system. A broadly defined benefit could be referring a patient onto a palliative care pathway. To date, only limited empirical data is available on TLT in intensive care. These procedures should be standardised and subjected to a rigorous substantive assessment to determine whether the expected benefits can be achieved within the complex realities of intensive care practice. One proposed framework structures these conversations around four elements: the Truth about prognostic uncertainty, the Interval of time agreed, the Measurement of improvement to be used, and whether to End or extend treatment at review [47].

To develop the best possible systems for predicting treatment outcomes, it is essential to take a more in-depth look at the analysis of the available data. Conventional scores derived from a single early assessment window describe risk at one moment and cannot, by design, reflect how it evolves during the ICU stay [54,55]. Large historical databases, containing millions of clinical cases in medical repositories, can be analysed with more flexible mathematical algorithms, interpreted in the context of the probability of a given endpoint occurring, whilst taking alternative scenarios into account [65]. Today, we refer to such processes as machine learning or deep learning, commonly summarised under the term ‘artificial intelligence’. It should be pointed out, however, that the results of such statistical experiments should not be uncritically accepted as true. To enhance their reliability, they must be interpreted by a human. At present, ICU-focused artificial intelligence models remain predominantly retrospective, are reported almost exclusively in terms of discrimination while calibration and clinical utility are rarely addressed, and show little evidence of regulated, routine deployment as clinical decision support [66]. They should therefore not be used autonomously or as a sole basis for decisions on the intensity of therapy; their role is adjunctive and contingent on prospective validation, transparent reporting and appropriate clinical governance. This mirrors a longer-standing pattern: despite reasonably well-performing ICU risk scores having existed for fifty years, no current ICU guidelines incorporate them to drive specific clinical actions [65].

## 8. Conclusions

Prognostic scoring systems remain a useful part of intensive care practice, but their usefulness is narrower than their routine application suggests. The instruments reviewed here answer three different questions: how ill was this patient on admission, how are their organ systems responding to treatment, and what functional state did they leave the unit in; none of them was designed to answer the question most often asked of them, namely whether this individual patient should continue to receive intensive therapy. Admission scores such as APACHE II and SAPS II/3 were derived and validated for cohort-level comparison; SOFA was designed to describe organ dysfunction over time rather than to predict death; the functional instruments used at discharge measure outcome rather than forecast it. The weaknesses of the mortality prediction models among them—reliance on an initial assessment window, dependence on historical case mix, absence of local recalibration, and a tendency to overestimate mortality in some validation settings—largely reflect how they were built and are unlikely to be removed by further validation of the same design alone. Progress is therefore more likely to come from the dynamics of change than from more precise measurement at a single moment: from trajectory-based models, from patient phenotyping and biomarker integration, and from standardised time-limited trials that convert prognostic uncertainty into a defined and revisable plan of care. Machine learning may accelerate this, but only under reporting and validation standards it has so far largely escaped. Until then, the appropriate role of a prognostic score is to open a structured conversation within a multidisciplinary team, and never to close one.

## Figures and Tables

**Table 1:** APACHE II, SAPS II and SAPS 3 scoring systems.

Scale	APACHE II	SAPS II	SAPS 3
Number of variables	14	17	20
Physiological variables	Temperature (core) Mean arterial pressure Heart rate (ventricular response) Respiratory rate (ventilated or nonventilated) Oxygenation (AaDO_2_ or PaO_2_) Arterial pH Serum sodium Serum potassium Serum creatinine (doubled for acute renal failure) Haematocrit White blood cell count Glasgow Coma Scale (GCS)	Heart rate Systolic blood pressure Body temperature PaO_2_/FiO_2_ ratio (if ventilated) Urine output Blood urea nitrogen (BUN) White blood cell count Serum potassium Serum sodium Serum bicarbonate Total bilirubin Glasgow Coma Scale	Glasgow Coma Scale (GCS) Systolic blood pressure Heart rate Body temperature Oxygenation and ventilation status (PaO_2_/FiO_2_ ratio and ventilation use) Total bilirubin Serum creatinine Leukocytes (white blood cell count) Platelets pH (hydrogen ion concentration)
Comorbidities	Heart failure class IV Cirrhosis Chronic lung disease Dialysis-dependent Immunocompromised	Acquired immunodeficiency syndrome (AIDS) Metastatic cancer Haematological malignancy	Cancer therapy Haematological cancer Chronic heart failure (NYHA class IV) Cirrhosis AIDS Metastatic cancer
Additional parameters	Age	Age Type of admission (scheduled surgical, unscheduled surgical, or medical)	Age Length of stay before ICU admission (days) Intrahospital location before ICU admission Use of vasopressors before ICU admission Planned or unplanned ICU admission Surgical status at ICU admission Anatomic site of surgery Presence of infection at ICU admission and place acquired (nosocomial or respiratory) Reason(s) for ICU admission (categorised by cardiovascular, neurological, digestive, or hepatic conditions)
Predicted outcome	In-hospital mortality	In-hospital mortality	In-hospital mortality
Comments	Traditionally validated to estimate total in-hospital mortality rather than strictly isolated ICU death, though it strongly correlates with both [25,26]		

**Table 2:** Variables used by the SOFA and SOFA-2 scales to assess the severity of organ dysfunction [12,39].

Organ System	SOFA	SOFA-2
Brain	Glasgow Coma Scale (GCS)	Glasgow Coma Scale (GCS); in sedated patients, the last GCS recorded before sedation Best motor response when the full GCS cannot be assessed Drug treatment for delirium (1 point even if GCS is 15)
Respiratory	PaO_2_/FiO_2_ Mechanical ventilation status	PaO_2_/FiO_2_ SpO_2_/FiO_2_ (only if PaO_2_/FiO_2_ is unavailable and SpO_2_ < 98%) Advanced ventilatory support: high-flow nasal cannula (HFNC) Continuous positive airway pressure (CPAP), bilevel or non-invasive ventilation (NIV) Invasive mechanical ventilation or long-term home ventilation Extracorporeal membrane oxygenation (ECMO)
Cardiovascular	Mean arterial pressure (MAP) Dopamine Epinephrine, norepinephrine, dobutamine	Mean arterial pressure (MAP) Combined norepinephrine and epinephrine dose Any other vasopressor or inotrope Dopamine (separate cut-offs when used alone) Mechanical circulatory support (MCS)
Kidney	Creatinine Urine output	Creatinine Urine output Renal replacement therapy (RRT), received or indicated
Haemostasis	Platelet count	Platelet count
Liver	Bilirubin	Bilirubin

**Table 3:** The Barthel index for activities of daily living.

Activity	Assessment Criteria and Definition	Points
1. Feeding	Unable/Dependent	0
	Needs help (e.g., cutting food, spreading butter, using condiments)	5
	Independent (food is within reach)	10
2. Bathing	Dependent	0
	Independent (washes self completely in bath or shower)	5
3. Grooming	Needs help with personal hygiene	0
	Independent (cleans face, hands, teeth, shaves, combs hair)	5
4. Dressing	Dependent	0
	Needs help but can do at least half of the work unaided	5
	Independent (includes buttons, zippers, laces, fasteners)	10
5. Bowels	Incontinent (or requires enemas)	0
	Occasional accident (maximum once per week)	5
	Continent	10
6. Bladder	Incontinent (or catheterised and unable to manage)	0
	Occasional accident (maximum once per 24 h)	5
	Continent (for more than 7 days)	10
7. Toilet use	Dependent	0
	Needs some help (for balance, clothing, or wiping)	5
	Independent (getting on/off, wiping, arranging clothes)	10
8. Transfers (bed to chair)	Unable (no sitting balance)	0
	Major help (1–2 people physical help, can sit up)	5
	Minor help (verbal supervision or minor physical guidance)	10
	Independent	15
9. Mobility (on level surfaces)	Immobile or steps < 50 yards	0
	Wheelchair independent (propels self > 50 yards)	5
	Walks with help (physical or verbal aid of 1 person > 50 yards)	10
	Independent (may use assistive aids like a cane > 50 yards)	15
10. Stairs	Unable	0
	Needs help (physical aid, verbal guidance, or carrying a cane)	5
	Independent (can safely go up and down)	10
Interpretation	0–20: Total dependency; 21–60: Severe dependency; 61–90: Moderate dependency; 91–99: Slight dependency; 100: Complete independence	

**Table 4:** CPC and GOSs for assessing neurological impairment.

Score	Cerebral Performance Category (CPC)	Glasgow Outcome Scale (GOS)
1	Best outcome: Conscious, alert, normal life, minor deficits	Death: Resulted in the patient’s death
2	Moderate disability: Conscious, independent in daily life, sheltered work	Vegetative state: Unresponsive, no conscious awareness
3	Severe disability: Conscious, dependent on others for daily support	Severe disability: Conscious, dependent for daily care
4	Coma/Vegetative state: Unconscious, no awareness	Moderate disability: Independent despite disability
5	Brain death/Dead: Dead by traditional criteria	Good recovery: Resumed normal life activities

## Data Availability

No new data were created or analysed in this study. Data sharing is not applicable to this article.

## Abbreviations

APACHE	Acute Physiology and Chronic Health Evaluation
SAPS	Simplified Acute Physiology Score
MPM	Mortality Probability Model
CFS	Clinical Frailty Scale
FSS-ICU	Functional Status Score for the ICU
SOFA	Sequential Organ Failure Assessment
MODS	Multiple Organ Dysfunction Score
TISS-28	Therapeutic Intervention Scoring System (28-item version)
BI	Barthel Index
ADL	Activities of Daily Living (Katz Index)
GCS	Glasgow Coma Scale
CPC	Cerebral Performance Category
GOS	Glasgow Outcome Scale
ICU	Intensive Care Unit
AUROC	Area Under the Receiver Operating Characteristic curve
MOF	Multi-Organ Failure
TLT	Time-Limited Trial
MACCE	Major Adverse Cardiac and Cerebrovascular Events
NfL	Neurofilament light
RRT	Renal Replacement Therapy
VIS	Vasoactive-Inotropic Score
AIDS	Acquired Immunodeficiency Syndrome
BUN	Blood Urea Nitrogen
CPAP	Continuous Positive Airway Pressure
ECMO	Extracorporeal Membrane Oxygenation
HFNC	High-Flow Nasal Cannula
MAP	Mean Arterial Pressure
MCS	Mechanical Circulatory Support
NIV	Non-Invasive Ventilation
NYHA	New York Heart Association
